# Mapping Alterations to the Endogenous Elemental Distribution within the Lateral Ventricles and Choroid Plexus in Brain Disorders Using X-Ray Fluorescence Imaging

**DOI:** 10.1371/journal.pone.0158152

**Published:** 2016-06-28

**Authors:** Brittney R. Lins, Jake M. Pushie, Michael Jones, Daryl L. Howard, John G. Howland, Mark J. Hackett

**Affiliations:** 1 Department of Physiology, University of Saskatchewan, Saskatoon, SK, Canada; 2 College of Medicine, University of Saskatchewan, Saskatoon, SK, Canada; 3 Australian Synchrotron, Clayton, Victoria, Australia; 4 ARC Centre of Excellence in Advanced Molecular Imaging, La Trobe Institute for Molecular Sciences, La Trobe University, Victoria, Australia; 5 Department of Chemistry, Curtin University, Perth, WA, Australia; Argonne National Laboratory, UNITED STATES

## Abstract

The choroid plexus and cerebral ventricles are critical structures for the production of cerebral spinal fluid (CSF) and play an important role in regulating ion and metal transport in the brain, however many aspects of its roles in normal physiology and disease states, such as psychiatric illness, remain unknown. The choroid plexus is difficult to examine *in vivo*, and *in situ ex vivo*, and as such has typically been examined indirectly with radiolabeled tracers or *ex vivo* stains, making measurements of the endogenous K^+^, Cl^−^, and Ca^+^ distributions unreliable. In the present study, we directly examined the distribution of endogenous ions and biologically relevant transition metals in the choroid plexus and regions surrounding the ventricles (ventricle wall, cortex, corpus callosum, striatum) using X-ray fluorescence imaging (XFI). We find that the choroid plexus was rich in Cl^−^ and Fe while K^+^ levels increase further from the ventricle as Cl^−^ levels decrease, consistent with the known role of ion transporters in the choroid plexus CSF production. A polyI:C offspring displayed enlarged ventricles, elevated Cl^−^ surrounding the ventricles, and intraventricular calcifications. These observations fit with clinical findings in patients with schizophrenia and suggest maternal treatment with polyI:C may lead to dysfunctional ion regulation in offspring. This study demonstrates the power of XFI for examining the endogenous elemental distributions of the ventricular system in healthy brain tissue as well as disease models.

## Introduction

The choroid plexus is the main cellular structure within the ventricular system, and has long been considered a filter or “kidney of the brain”. Despite a relatively simple cellular composition, the chemical and physiological roles of the choroid plexus are not fully known. It is widely accepted that one function of the choroid plexus is production of cerebral spinal fluid (CSF) and regulation of CSF volume [1, 2]. Several physiological pathways in the choroid plexus regulate CSF production, including ion transport and osmotic water transfer [1–4]. Cells of the choroid plexus system are rich in ion-transporters, particularly the Na^+^,K^+^ATPase transporter, which is the main regulator of CSF volume [3, 4]. Through regulation of intracellular and extracellular Na^+^ and K^+^ concentration, the choroid plexus creates a gradient in Cl^−^ concentration [1–3]. The Cl^−^ concentration within the CSF, therefore, is maintained at a higher level than in plasma, allowing water to follow the concentration gradient and transfer from the plasma through the epithelium of the choroid plexus and into the CSF, thus, increasing CSF volume [1–4].

In addition to CSF production and CSF volume regulation, the choroid plexus is increasingly recognized as a source of ion and metal transport into the brain. Radiolabelled ion tracer studies have demonstrated that ions, such as Cl^−^, cross the choroid plexus—ventricle—brain barrier at a rate up to 200x greater than crossing from plasma across the blood brain barrier [1, 5]. In addition, studies of metal ion transport reveal that the choroid plexus is enriched in specific Fe, Cu and Zn transporters, as well as non-specific divalent metal ion transporters [6–8]. Therefore, it is likely that the choroid plexus not only performs roles as a filter system and site for CSF production and regulation, but may also hold a role as a supplier of ions and transition metals to the brain.

Recent studies demonstrate that activity within the choroid plexus is influenced by cells within the brain parenchyma to a much greater extent than previously thought [9]. For example, increased serotonin release by brain cells increases protein kinase C mediated phosphorylation of Na^+^,K^+^ATPase, which decreases its activity and thus decreases CSF production [3]. It is well established that altered levels of serotonin or serotonin receptors occur during neurological disorders such as depression [10], anxiety [11], and schizophrenia [12]. Further, patients suffering from schizophrenia have enlarged ventricles [13–16] which often contain micro-calcifications, indicative of abnormal CSF production and disturbed ion homeostasis [17–20]. This suggests that altered serotonin levels during neurological disease may significantly impact function of the choroid plexus causing downstream effects such as abnormal CSF production, ventricle swelling and altered ion and transition metal ion homeostasis. As Cl^−^ and Ca^2+^ have important roles as neurotransmitters for GABAergic and glutamatergic neurons and inter-neurons, altered brain ion homeostasis in response to altered choroid plexus function may exacerbate or worsen neurological disorders. However, this has been little studied.

A primary reason for the lack of information available on the downstream effects of altered choroid plexus function on ion homeostasis is the lack of a suitable imaging technique. Traditionally, the levels of ions and metals within brain tissue would be quantified by tissue micro-dissection and elemental assay (i.e., atomic absorption spectrometry, or mass spectrometry). However, micro-dissection of the ventricle system and isolation of the fragile choroid plexus from CSF is essentially impossible, and therefore bulk elemental analysis of both these structural compartments is rarely reported. At present, the majority of knowledge on the distribution of diffusible ions within the choroid plexus and surrounding brain parenchyma is derived from immuno-histochemical analysis of the distribution of the respective ion transporters, or radiolabelled imaging of exogenously administered ions [21–23]. A range of fluorescence dyes exists for in vivo Ca^2+^ imaging, however, laser penetration depth is a limiting factor and these experiments cannot be performed on deeper brain structures such as the choroid plexus and ventricle system. In addition, due to the highly mobile and diffusible nature of ions such as Cl^−^, K^+^ and Ca^2+^, *ex vivo in situ* staining for these ions in tissue sections is not possible as immersion of the tissue in any fixation or staining media re-distributes or completely removes these ions from the tissue [24]. Therefore, a technique capable of *in situ* imaging the endogenous elemental distribution at the cellular or sub-cellular level in *ex vivo* tissue sections, would be of great benefit to studying choroid plexus regulation of brain ion homeostasis, particularly in neurological disorders or disease.

One technique that holds great promise for direct *in situ* imaging of endogenous elements in *ex vivo* brain tissue sections is X-ray fluorescence imaging (XFI) [25, 26]. The technique can routinely spatially resolve elemental distribution at the micron level. Further, the use of modern synchrotron lightsources and advanced detector systems enables rapid image collection times with low detection limits, enabling application of this technique to challenging scientific questions pertinent to the biological sciences. The effect of sample preparation on the elemental composition of brain tissue has been studied and optimized, and XFI analysis of flash frozen brain tissue without any form of chemical fixation or perfusion has been shown to reveal the elemental distribution as close as possible to the *in vivo* state [24, 27].

XFI has found numerous applications to the field of neuroscience and elemental maps of the choroid plexus and ventricle systems have previously been reported in several studies. Specifically, XFI has been used to study the location of Cu in ventricle walls and the sub-ventricular zone [28, 29], as well as to investigate Mn transport from the choroid plexus into the brain [30]. However, as these previous studies employed chemical fixation of the brain tissue, elemental maps of the distribution of diffusible ions that reflect the *in vivo* state were not obtained. Further, due to the higher energies of the K shell emission lines of transition metals, experiments can often be performed in air, however, this results in substantial attenuation of K shell emission lines of biologically important ions such as Cl^−^ and K^+^. Performing XFI experiments in a He environment reduces the low energy attenuation and allows more accurate detection of Cl^−^ and K^+^ ions, in addition to transition metals.[31] In this investigation, we demonstrate the immense potential of XFI elemental mapping to study the function of choroid plexus in maintaining ion and transition metal homeostasis within the brain. Using XFI we obtained quantitative elemental content (P, S, Cl, K, Ca, Fe, Cu, Zn) within the choroid plexus, sub-ventricular zone and surrounding brain tissue regions. To the best of our knowledge, no previous study using XFI or any other technique has quantified the elemental content of the choroid plexus and ventricle system or compared the elemental levels with those of the surrounding brain tissue. To further demonstrate the potential of incorporating XFI in future studies to correlate altered choroid plexus function with altered brain ion homeostasis in neurological disease or disorders, we report a case study (single animal) highlighting the elemental alterations that may occur within the choroid plexus and ventricular system in an animal model of schizophrenia, which is characterized by altered brain serotonin homeostasis and enlarged ventricles.

## Materials and Methods

### Animals

Time pregnant Long-Evans rats (Charles River Laboratories, Quebec, Canada) arrived at the animal holding facility on gestational day (GD7). Dams were single housed in standard polypropylene cages in a temperature controlled (21°C) colony room on a 12/12 h light/dark cycle with food (Purina Rat Chow) and water available ad libitum. Experimental procedures were carried out during the light phase (lights on at 07:00 h). All experimental procedures were conducted in accordance with the Canadian Council on Animal Care and were approved by the University of Saskatchewan Animal Research Ethics Board. No animals utilized for this research became ill or died prior to the experimental endpoint.

### Maternal Treatments

On GD15, dam weight and rectal temperature were recorded. Dams were anesthetized with isoflurane (5% induction, 2.5% maintenance) for <10 minutes to receive a single intravenous (i.v.) injection of either saline or polyI:C (4 mg/kg, high molecular weight, InVivoGen, San Diego, CA, USA). Aside from additional maternal weight and temperature monitoring 8, 24, and 48 h following treatment, dams were left undisturbed and allowed to deliver naturally. On postnatal day (PND) 1, litters were weighed, sexed, and culled to a maximum of 10 (6 males where possible). On PND21, pups were weaned and housed in same-sex groups of 2 or 3 in standard housing as previously described. Offspring from these litters were randomly selected for synchrotron imaging with each rat coming from a separate litter. Culled pups and dams were euthanized with deep isoflurane anesthesia followed by cervical dislocation.

### Tissue Collection and Sample Preparation

At PND60, four offspring (*n* = 4) of four saline-treated dams (one offspring from each dam) and a single offspring (*n* = 1) of a polyI:C-treated dam, were deeply anesthetized with isoflurane and sacrificed by decapitation. The non-dissected head was immediately immersed in liquid nitrogen and stored at -80°C. On dry ice, rat heads were chiseled along the sagittal plane into equal halves and brains were further exposed using a Dremmel blade. Cryo-sections (20 μm) were cut at -18°C, at two different brain locations at -0.5 mm and -3.6 mm anterior to bregma respectively. Sections were cut from both left and right hemispheres, i.e., four tissue sections per animal. Following tissue sectioning, the sections were melted onto the relevant substrate and air-dried. Tissue sections were kept at ambient room temperature in a desiccated sealed container until analysis (< 2 weeks between sectioning and anlaysis). Two tissue sections were mounted on Si_3_N_4_ substrate (10x10 mm silicon frame, 200 μm thick, 5x5 mm Si_3_N_4_ membrane, 200 nm thick, Norcada, Edmonton, AB, Canada) for imaging at the X-ray fluorescence microscopy beamline at the Australian Synchrotron, the other two sections were mounted on thermanox plastic slides for analysis at the Stanford Synchrotron Radiation Lightsource. Adjacent tissue sections were also cut at a thickness of 10 μm and were mounted on microscope slides for staining with cresyl violet.

To the best of our knowledge a direct comparison of elemental distribution in air-dried tissue sections cut from flash-frozen brains versus cryosections analyzed frozen and hydrated with a cryostream has not been performed. Therefore, the extent to which thawing of the tissue section during air-drying alters elemental distribution is not known. We believe it is unlikely that re-distribution on the order of microns would occur due to the thin nature of the sections and their rapid air-drying time. However, sub-micron and sub-cellular redistribution cannot be discounted, but would not be detected at the spatial resolution used in this study. Although studies comparing the elemental distribution in air-dried tissue sections and frozen hydrated tissue sections cut from flash frozen brain tissue have not been completed, we have previously analyzed the speciation and bulk levels of sulfur in such an experiment.[32] The results revealed that a subtle increase in thiol oxidation is observed in both air-dried and freeze dried tissue sections relative to tissue sections analyzed fully hydrated and frozen under a cryostream.[32] The levels of taurine, a small mobile diffusible molecule were identical in all three sample preparations, suggesting that bulk re-distribution does not occur during air-drying of semi-thin brain tissue.[32]

### Synchrotron Imaging

Elemental mapping of tissue sections generated from the right brain hemisphere was performed at the XFM beamline at the Australian Synchrotron.[33] A monochromatic incident beam of 12.7 keV was focused to a 2 μm x 2 μm spot with a Kirkpatrick–Baez mirror pair. X-ray emission from the sample were collected in event-mode using the low-latency, 384-channel Maia XRF detector array.[34] Data was collected with the sample orientated normal to the incident beam with the detector positioned in backscatter geometry. Data was collected with the sample under a Helium purge to facilitate detection of low Z elements, as previously reported.[31] The sample was raster scanned through the beam with an effective dwell time of 0.1 ms and an effective step (pixel) size of 1 μm. Elemental foils (Micromatter, Canada), were scanned in the same geometry and used as references for elemental quantification. Elemental maps were reconstructed from the full emission spectra with GeoPIXE v6.6j. (CSIRO, Australia), which uses a linear transformation matrix for spectral deconvolution.[35] Quantification was performed with calibration against elemental foils of known composition. The composition and density of the Si_3_N_4_ substrate and the approximate composition of the sample were incorporated in the quantification process. The composition of the tissue sample was assumed to approximate that of a dried organic material (C_22_H_10_N_2_O_4_), with a density of 1.42 g cm^-3^, as previously used by others.[36] Due to the small thickness of the sample (20 μm when cut) and the fact that the major component of the sample by weight, water, is removed during air-drying of the tissue section, self-absorption effects are negligible.[37] The elemental areal densities obtained in this investigation are in direct agreement with those previously reported by other research groups using XFI elemental analysis of rat brain sections at a similar thickness and with a similar sample preparation method.[38–41] Data was outputted from GeoPIXE as tiff files displaying the elemental concentration per pixel in ng cm^-2^, which was then converted to μg cm^-2^, and then imported into ImageJ v1.48. All regions of interest and the average elemental concentration were calculated from the raw images using ImageJ. A 3 point moving average was applied to decrease the appearance of noise and enhance image contrast, for images presented in the manuscript.

### Data Analysis

Data available in S1 File. All results are represented as group means ± SEM. Elemental concentrations of P, S, Cl, K, Ca, Fe, Cu, and Zn in each of the choroid plexus, corpus callosum, striatum, cortex, and ventricle wall were calculated from regions of interest drawn using ImageJ software. One-way Repeated Measures ANOVAs (SPSS version 23) were used to compare the concentrations of each element among the 5 areas assessed in the saline offspring. Post-hoc tests were calculated using Tukey’s HSD. The polyI:C offspring was not included in statistical analysis but used to illustrate the potential for using synchrotron light to study models of psychiatric illness.

### Analysis at the Stanford Synchrotron Radiation Lightsource

To replicate the results observed from analysis at the Australian Synchrotron, XFI elemental maps were collected from tissue sections of the left brain hemisphere, using synchrotron light at beamline 10–2 at the Stanford Synchrotron Radiation Lightsource (SSRL) (http://www-ssrl.slac.stanford.edu/beamlines/bl10-2/). The energy of the incident X-ray beam was 13450 eV, and the incident X-ray intensity was measured using a nitrogen gas-filled ion chamber (I0). A 50 μm pin-hole aperture was used to define the beam size on the sample. The sample was mounted at 45° to the incident beam and raster scanned through the beam using a “fly” scan or “rapid” scan mode. A single-element Vortex^®^ silicon drift detector at 90° to the incident beam collected single channel (SCA) X-ray fluorescence spectra from the brain tissue. The detector readout was synchronized to the stage movement speed, and data collected continuously, such that the full emission spectrum was collected every 200 ms, for an average stage movement of 30 μm. SCA fluorescence data was not converted to quantitative maps as measurements were performed under ambient laboratory atmosphere, where Ar absorption is not negligible for the emission lines of light elements such as P, S, Cl, K and Ca. The SCA fluorescence maps were normalized to I0, and visualized using microtool kit software (http://smak.sams-xrays.com/).

## Results and Discussion

### Validation of XFI to Study the Elemental Composition of the Choroid Plexus, Sub-Ventricular Zone and Surrounding Brain Regions

**Fig 1:**
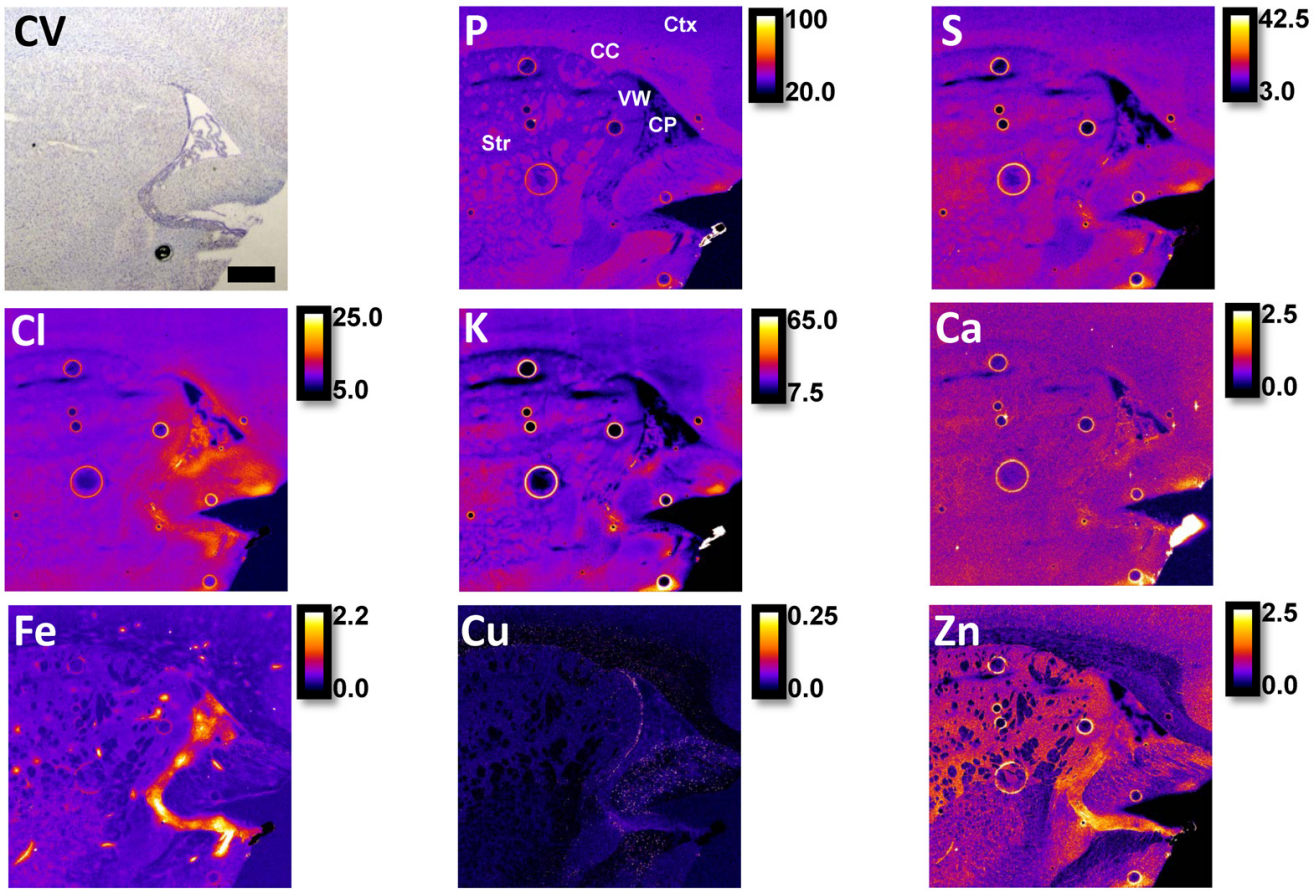
A comparison of routine cresyl violet (CV) histology and elemental maps (P, S, Cl, K, Ca, Fe, Cu, Zn) revealed by XFI for a representative saline control rat at PND60. The brain regions of interest are choroid plexus (CP), corpus callosum (CC), striatum (Str), cortex (Ctx), and ventricle wall (VW). All examined elements are observed in the choroid plexus with notably high levels of Fe and Cl relative to other brain regions. Scale bar = 500 μm, intensity units are μg cm^-2^.

**Fig 2:**
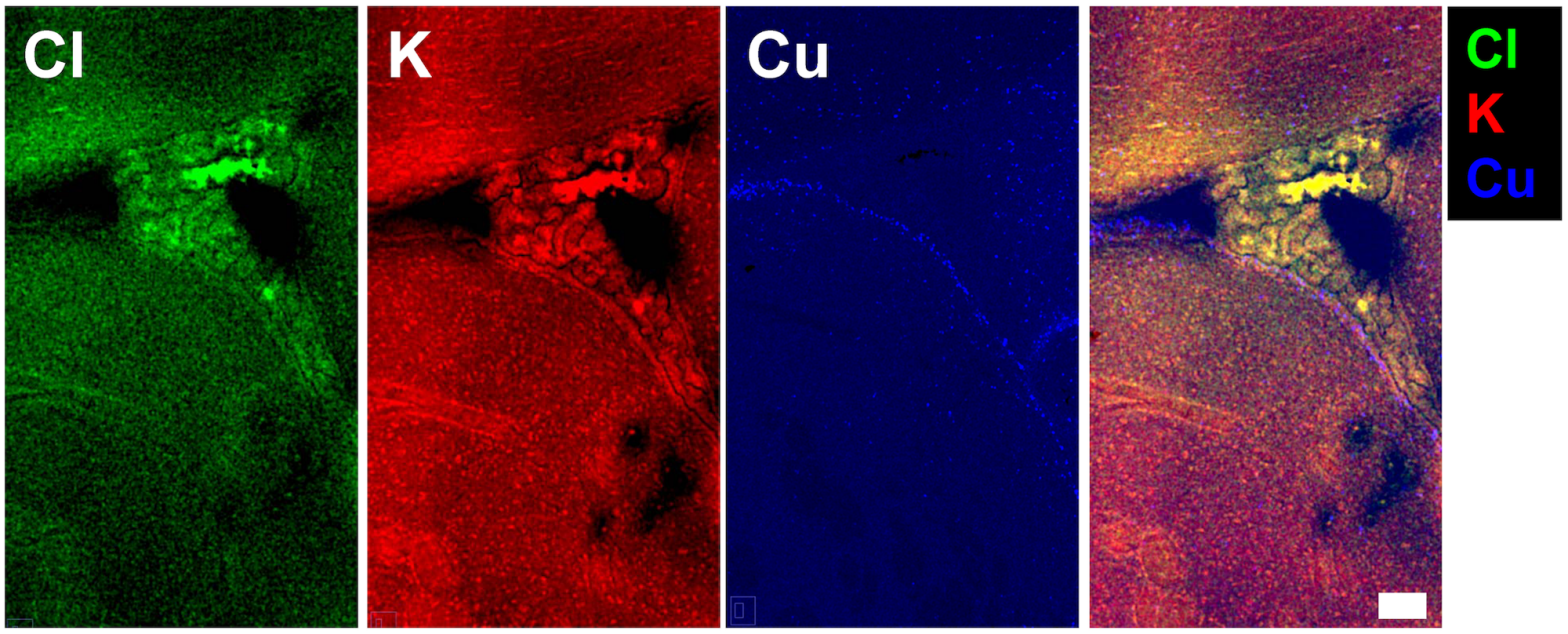
An enlarged view of elemental maps of Cl^−^, K^+^ and Cu in the choroid plexus and ventricle wall with tri-colour overlay. Numerous Cu hot spots mark the ventricle wall, and Cl^−^ and K^+^ co-localize with the choroid plexus epithelium. The tri-colour overlay highlights that K^+^ is at lower concentration outside the choroid plexus epithelium, while Cl^−^ is still abundant. Scale bar = 100 μm.

**Fig 3:**
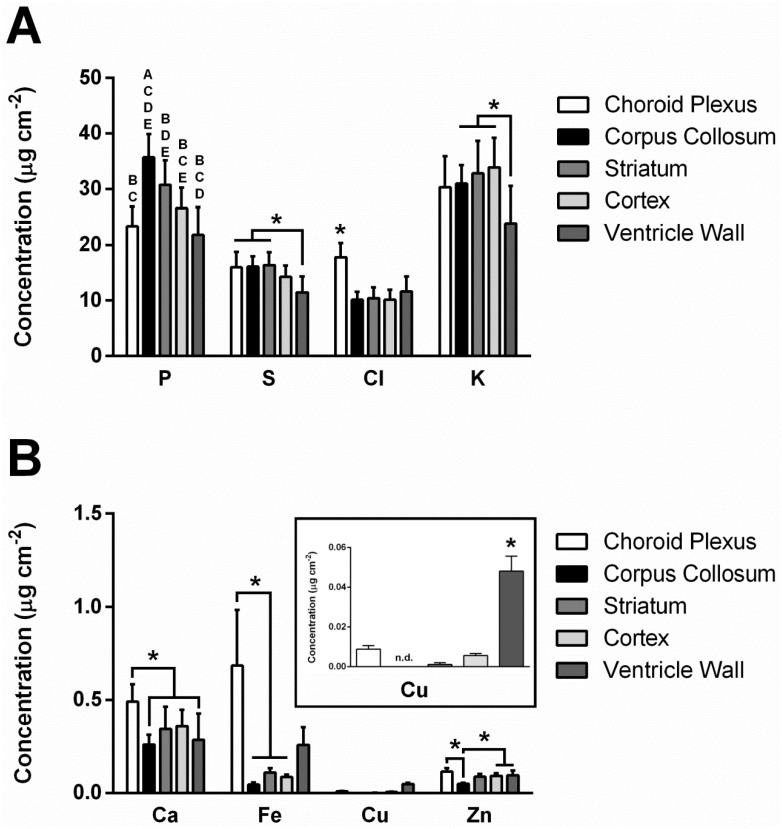
Elemental quantification performed with XFI of distinct brain regions (choroid plexus, corpus callosum, striatum, cortex, and ventricle wall). One-way repeated measures ANOVA revealed significant differences in all elements as a factor of brain region (p<0.05). Tukey’s HSD was used to calculate post hoc tests. [**A**] Elemental concentrations for P, S, Cl, and K. A letter system is used to denote significant differences in P across brain regions. A = significantly different from choroid plexus, B = significantly different from corpus callosum, C = significantly different from striatum, D = significantly different from cortex, E = significantly different from ventricle wall. For all other elements, significant differences are indicated with an asterisk (*). [**B**] Elemental concentrations for Ca, Fe, Cu, and Zn. Significant differences are indicated with an asterisk (*).

The distribution of elements (P, S, Cl, K, Ca, Fe, Cu, Zn) within the choroid plexus, ventricle system, and surrounding brain tissue, revealed by XFI, are presented in a representative brain tissue section (Fig 1). Visual observation of the elemental maps highlights that all the studied elements are present within the choroid plexus, which is in agreement with the known abundance of ion transporters, non-specific divalent cation transporters and specific metal transporters within the choroid plexus [1, 2, 4, 6–8]. Specifically, the choroid plexus visually appears to contain the greatest concentration of Cl^−^ and Fe, as well as high concentrations of other elements, such as K^+^ and Ca^2+^. Cl^−^and K^+^ were observed in high concentration within the choroid plexus epithelium, however K^+^ was observed in relatively low concentration outside of the epithelium, while Cl^−^ was still abundant (Fig 2). To confirm these observations, tissue sections were analyzed from 4 animals and the average elemental concentration determined from specific brain tissue regions (choroid plexus, ventricle wall, striatum, corpus callosum and cortex). As shown in Fig 3, statistical analysis using repeated measures ANOVA confirmed that the choroid plexus contains the highest concentration of Cl^−^ and Fe relative to the other brain regions studied. Additionally, each element studied was found to have significant differences among the regions of interest (Fig 2; P (F_(4,12)_ = 34.951; *p* <0.001), S (F_(4,12)_ = 10.872; *p* <0.01), Cl (F_(4,12)_ = 17.512; *p*<0.05), K (F_(4,12)_ = 6.633; *p*<0.01), Ca (F_(4,12)_ = 4.613; *p*<0.05), Fe (F_(4,12)_ = 4.275; *p*<0.05), Cu (F_(4,12)_ = 34.951; *p*<0.001), Zn (F_(4,12)_ = 7.578; *p*<0.001); results of post hoc are illustrated).

The significantly elevated levels of Fe observed within the choroid plexus relative to other brain regions is consistent with the high level of blood flow to the choroid plexus epithelial cells, and the high mitochondria content of choroid plexus epithelial cells [1, 2, 4, 42]. The epithelial cells of the choroid plexus are rich in ion transporters, in particular Na^+^,K^+^ATPase, which acts to create a strong Cl^−^ concentration gradient, and likely accounts for the significantly increased Cl^−^ concentration observed within the choroid plexus relative to other brain regions [1, 3, 4, 22].

**Fig 4:**
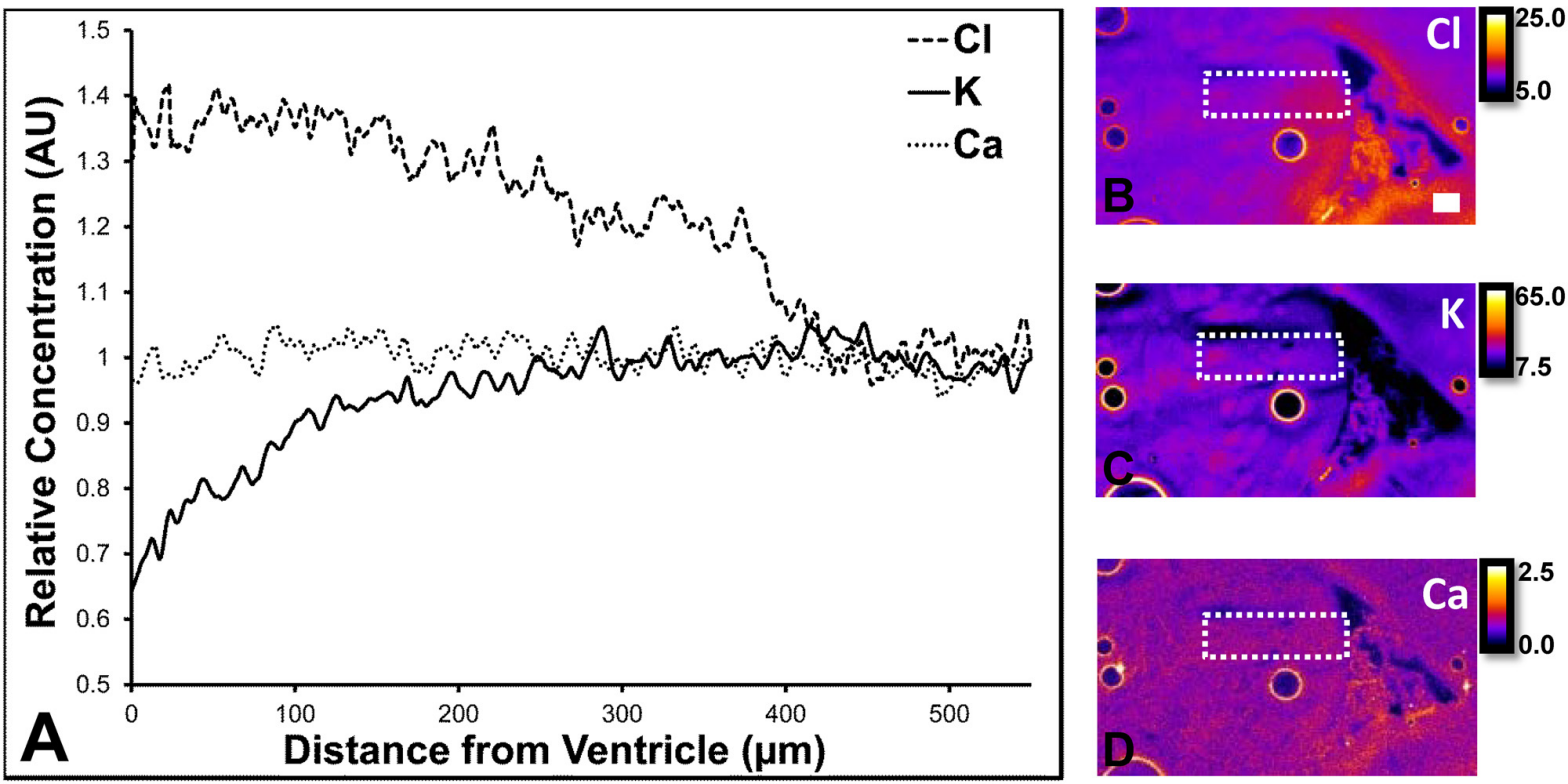
[**A**] Radial profiles of Cl^−^, K^+^, and Ca^2+^ concentrations across the ventricle wall, sub-ventricular zone, and surrounding striatum. [**B–D**] XFI elemental maps of Cl^−^, K^+^, and Ca^2+^, respectively, illustrating the region from where radial profiles were calculated (white dashed box). Scale bar = 100 μm, intensity units are μg cm^-2^.

In addition to the calculation of the average elemental concentration for specific brain regions, XFI revealed the endogenous concentration gradient of Cl^−^, K^+^, and Ca^2+^ across the ventricle system and surrounding brain tissue. As can be seen in Fig 4, the Cl^−^ concentration is greatest along the ventricle wall and within the sub-ventricular zone, decreasing with distance from the ventricle wall, which is in strong agreement with the known transport of radiolabeled Cl^−^ from the blood stream into the brain parenchyma via the CSF and ventricle system. The K^+^ gradient is the inverse of the Cl^−^ gradient, with increasing K^+^ concentration observed with increasing distance from the ventricle wall. This is consistent with the known uptake and removal of K^+^ from the ventricle system and CSF by the choroid plexus epithelium [1, 3–5, 22, 23]. Despite the large variations in Cl^−^ and K^+^ concentration that can be induced by the normal function of the choroid plexus, it is has been established that the Ca^2+^ concentration is tightly regulated [1, 4]. Not surprisingly, XFI did not reveal a Ca^2+^ concentration gradient. To the best of our knowledge, these results are the first demonstration of endogenous ion concentration gradients, and are in agreement with the concentration gradients that have been proposed to exist based on studies using exogenously administered ions [5, 23].

### XFI Elemental Mapping Suggests Altered Choroid Plexus and Ventricle Ion Homeostasis in an Animal Model of Schizophrenia

**Fig 5:**
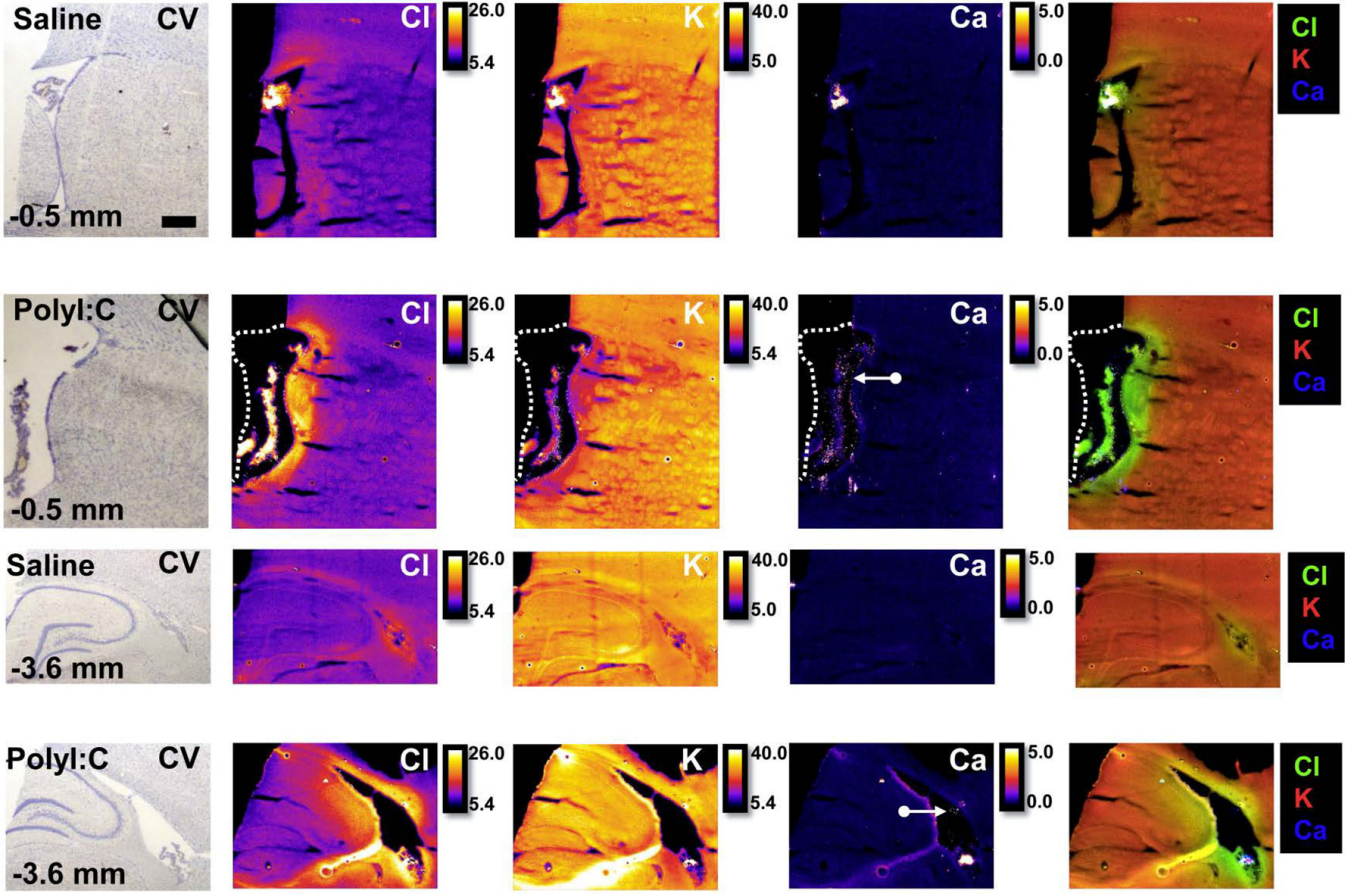
Routine cresyl violet (CV) histology and XFI elemental mapping (P, S, Cl, K, Ca, Fe, Cu, Zn) of the offspring (PND60) from a saline control rat and a polyI:C immune compromised rat. Images were collected from tissue sections -0.5 mm and -3.6 mm anterior to bregma. White arrows indicate the presence of numerous calcifications observed within the ventricles of the polyI;C offspring. Due to the increased swelling of the ventricle in the polyI:C offspring at bregma location -0.5mm, the medial side of the ventricle wall tore from the tissue section. A white dashed line shows the approximate location of the ventricle wall before it tore away during tissue sectioning. Scale bar = 500 μm, intensity units are μg cm^-2^.

**Fig 6:**
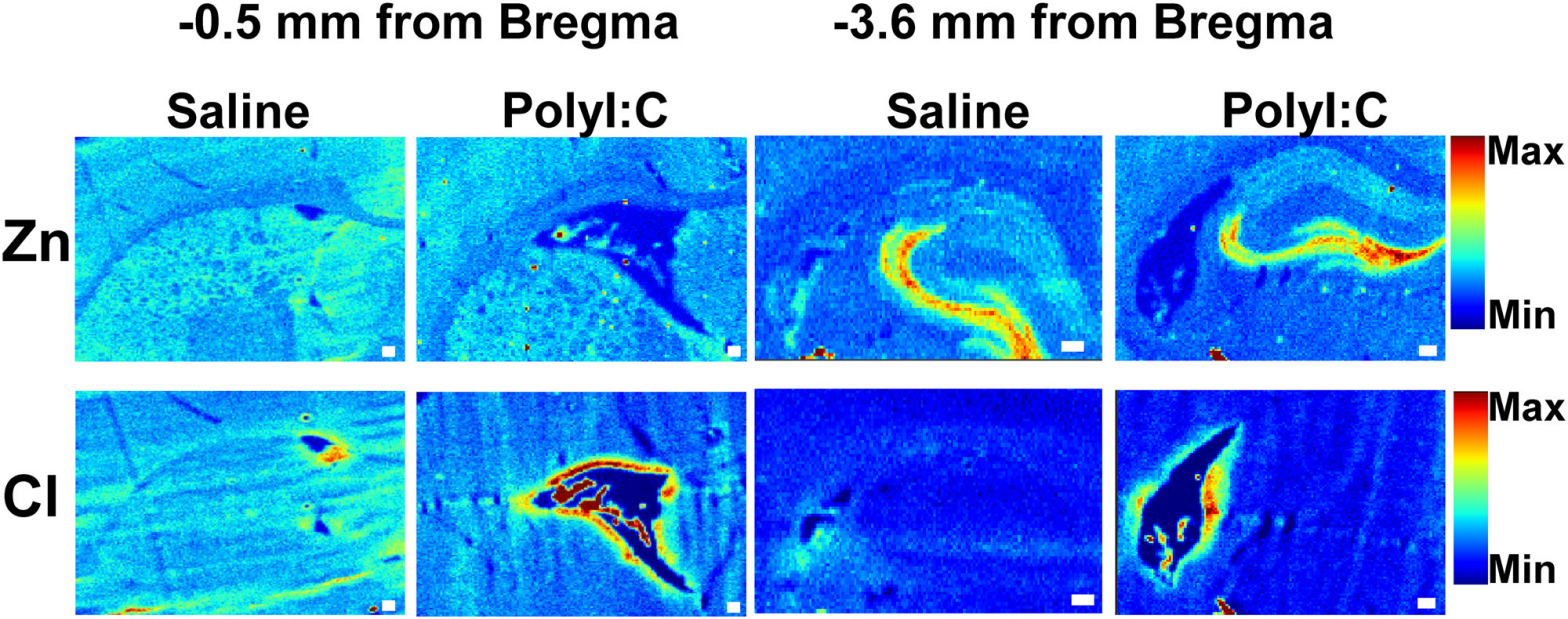
Replication of the Cl^−^ results from elemental mapping at the Australian Synchrotron was performed at the Stanford Synchrotron Radiation Lightsource using tissue sections from a similar anatomic plane collected from the opposite brain hemisphere to those presented in Fig 5. Elemental maps of Zn distribution are shown to highlight tissue anatomical structure (grey matter contains high Zn, white matter contains low Zn). Scale bar = 100 μm. Elemental maps are displayed on a relative scale, the scale is the consistent for all Zn maps, and a separate but consistent scale is used for all Cl^−^ maps.

**Fig 7:**
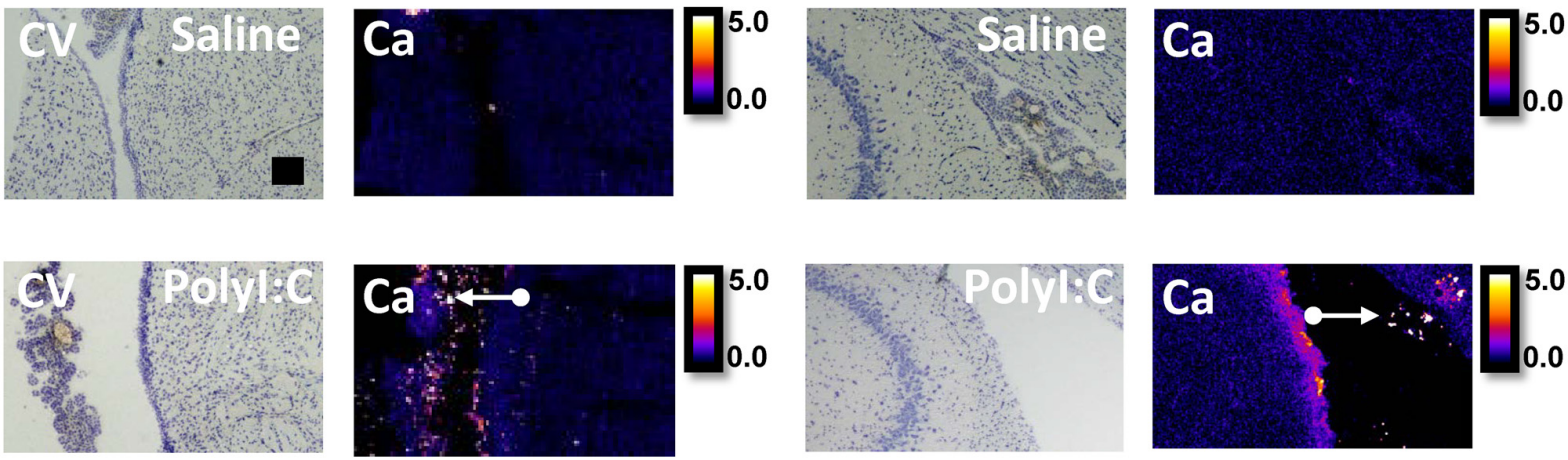
Cresyl violet (CV) histology and XFI elemental mapping of calcifications (white arrows) within ventricles. The first two columns correspond to images collected from tissue sections at a location of -0.5 mm anterior to bregma, and the last two columns correspond to images collected from tissue sections at a location of -3.6 mm anterior to bregma. Scale bar = 100 μm, intensity units are μg cm^-2^.

To demonstrate the potential of XFI elemental mapping to correlate altered choroid plexus function with disturbed ion transport and ion homeostasis within the choroid plexus in brain disorders, a case study (single animal) was performed using one offspring from a rat treated with polyI:C on GD15 of pregnancy, which is an animal model of schizophrenia.[43, 44] As can be seen in Fig 5, there is a substantial increase in Cl^−^ concentration within the choroid plexus and sub-ventricular zone in the polyI:C offspring relative to the control around the lateral ventricles at two different locations within the brain (ventricles adjacent to striatum 0.5 mm anterior to Bregma, and ventricles adjacent to hippocampus 3.6 mm to Bregma). To further demonstrate that these results are representative and reproducible in this polyI:C offspring, tissue sections from a similar anatomical plane were analyzed at the Stanford Synchrotron Radiation Lightsource which yielded a similar profile of elevated Cl^−^ around the lateral ventricle at both brain locations (Fig 6). In addition, data collected at the Australian synchrotron revealed decreased K^+^ levels in the sub-ventricular zone, and the ventricle appeared larger and contained numerous Ca deposits (Figs 5–7) in the polyI:C offspring relative to the control animals.

While the etiology of schizophrenia remains largely unknown, maternal inflammation and malnutrition [45–49], and peri/neonatal trauma such as hypoxia during birth [50–52], have been implicated with a higher incidence of schizophrenia in the offspring. While the incidence of schizophrenia among the general population is approximately 1%, maternal influenza during pregnancy increased the offspring’s risk by 3 to 7-fold.[53] The mechanisms underlying maternal infection as a risk factor for schizophrenia have been studied using the maternal immune activation (MIA) model in which mice or rats experience an immune challenge during pregnancy. Previous research using MIA via polyI:C in rats and mice demonstrated offspring have abnormalities in behaviour and cognitive performance as well as abnormalities in brain morphology such as enlarged ventricles which appeared post-puberty.[54–57] Thus, the model demonstrates face and construct validity as well as a neurodevelopmental ‘disease’ progression, similar to the manifestation of schizophrenia in humans. In human schizophrenia, micro-calcifications are often observed within the enlarged ventricles [58]; however, the presence of micro-calcifications has not previously been studied in the MIA animal model. Therefore, this study is the first to present evidence of micro-calcifications within enlarged ventricles in the polyI:C model of MIA-induced schizophrenia which correlates well with the human condition.

A possible explanation for the appearance of enlarged ventricles in human schizophrenia and in animal models, including the polyI:C animal analyzed in this study, could be excessive CSF production. Increased Cl^−^ levels and decreased K^+^ levels were observed in the subventricular zone and within the choroid plexus in the polyI:C animal. This may indicate increased Na^+^,K^+^,ATPase activity. Serotonin is an inhibitor of Na^+^,K^+^,ATPase activity, and decreased brain serotonin, as is found in human schizophrenia [12], as well as animal models of MIA [59–61], would result in a loss of this inhibitory action on Na^+^,K^+^,ATPase activity, giving rise to increased CSF volume and the resulting enlargement of the ventricles observed in schizophrenia. Although only one polyI:C offspring is presented in this manuscript, the results serve to demonstrate the ability to apply XFI to study alterations in the distribution of elements within the choroid plexus and surrounding brain tissue as a consequence of neurological disorders and neurological disease state.

## Conclusions

This study has used XFI elemental mapping to reveal for the first time the endogenous distribution of ions (Cl^−^, K^+^, and Ca^2+^) within the choroid plexus, ventricular system and surrounding brain tissue. The results of this study were in strong agreement with the ion distributions proposed by other studies using indirect techniques or endogenously administered ions. This is an important finding as it validates the use of XFI a new method to investigate ion homeostasis within the choroid plexus and ventricle system with the advantage of high spatial resolution direct imaging of the endogenous ions (i.e., no radiolabeled tracers are required). We also demonstrate the potential of this approach for studying models of psychiatric illness with a case study analysis in a rat model of schizophrenia. Our case study highlights increased Cl^−^, decreased K^+^ and micro-calcifications occur concomitant with increased ventricle size. The results of this study will now drive future investigations, incorporating XFI elemental mapping to further understand the role of the ventricle system in normal physiology and pathological mechanisms of neurological diseases. Specifically, the results of this case study will be statistically validated in a larger animal study, and further investigations will be performed to determine if micro-calcifications and altered Ca^2+^ homeostasis within the ventricles, and increased Cl^−^ concentration have downstream effects in the pathology of schizophrenia.

## Supporting Information

S1 File Data. Elemental concentrations summarized in Figs 3 and 4.
(XLSX)
